# 빛요법이 시설 노인의 수면 효율성에 미치는 효과: 체계적 고찰 및 메타분석

**DOI:** 10.7586/jkbns.25.071

**Published:** 2025-11-26

**Authors:** In Sun Cheon, Sun Hee Lee, Young Ran Chae

**Affiliations:** 1 Department of Nursing, Andong Science College, Andong, Korea; 1 안동과학대학교 간호학과; 2 Department of Nursing, Doowon Technical University, Anseong, Korea; 2 두원공과대학교 간호학과; 3 College of Nursing, Kangwon National University, Chuncheon, Korea; 3 강원대학교 간호대학 간호학과

**Keywords:** Aged, Residential facilities, Phototherapy, Sleep, Meta-analysis, 노인, 거주시설, 빛요법, 수면, 메타분석

## Abstract

**Purpose:**

This study aimed to systematically review the effects of light therapy on sleep efficiency in institutionalized older adults and to analyze effect sizes according to the characteristics of light therapy.

**Methods:**

A comprehensive literature search was conducted using RISS, KMBASE, KISS, NDSL, KISTI, NAL, PubMed, EMBASE, Cochrane Library, CINAHL, and ProQuest Global. Manual searches of reference lists were also performed. A total of 1,597 records were identified, and nine studies met the inclusion criteria and were included in the final analysis. Following quality assessment, all eligible studies were analyzed using RevMan and R software.

**Results:**

The overall effect size of light therapy on sleep efficiency was 0.67 (95% confidence interval [CI]: 0.13~1.21), indicating a beneficial effect. In subgroup analyses, studies using light intensity ≥ 5,000 lux showed a significant effect size of 1.58 (95% CI: 0.80~2.37). For studies with light exposure durations shorter than 1 hour (n = 4), the effect size was 1.40 (95% CI: 0.74~2.06). Similarly, studies with intervention periods shorter than 3 weeks (n = 4) demonstrated an effect size of 1.40 (95% CI: 0.74~2.06). However, heterogeneity was high in all analyses. In contrast, when light application timing was categorized as morning, afternoon, or all day, no significant effects were observed.

**Conclusion:**

Despite considerable heterogeneity, light therapy showed beneficial effects on sleep efficiency in institutionalized older adults. When applying light therapy, factors such as appropriate light intensity, exposure duration, and intervention period should be carefully considered.

## 서론

### 1. 연구의 필요성

인구의 노령화 현상이 세계적으로 빠른 속도로 진행되고 있는 가운데 우리나라의 경우 65세 이상의 노인인구는 2010년 10.8%의 고령화 사회에서 2020년 15.7%의 고령사회를 넘어 2025년에는 20.3%로 초고령사회에 진입하였으며, 경제협력개발기구(Organization for Economic Cooperation and Development) 국가 중 고령화 현상이 가장 빠르게 진행되고 있다[1]. 노인인구가 증가하면서 노인성 질환이 있는 노인도 함께 증가하고 있으며, 독립적인 일상생활이 어려운 노인들은 장기 요양시설이나 널싱홈 등 다양한 기관에서 돌봄을 받게 되고, 이에 따라 시설에 입소하는 노인인구는 점차 확대되고 있다[2].

시설 노인은 신체적, 정신적, 사회적 건강 수준이 전반적으로 취약하며, 고혈압, 당뇨병, 뇌혈관질환, 만성 요통, 관절염, 골절 등의 만성질환이 동반되어 있어 일상생활 수행 시 타인의 도움이 필요한 경우가 많다[3]. 또한 시설 환경에서 발생하는 다양한 자극으로 인해 적응에 어려움을 겪는 경우가 빈번하며, 이에 따라 건강하지 못한 수면 양상을 갖게 되어 수면장애를 경험하기도 한다[4]. 실제로 요양시설에 입소한 노인의 72.1%에서 수면 문제가 보고되었고[5], 시설 노인은 재가 노인보다 수면의 질과 양이 낮은 것으로 나타났다[6]. 수면 부족은 당뇨병, 고혈압, 심혈관 질환의 이환율 증가와 관련이 있으며[7], 피로감, 기억력 및 집중력 저하, 낙상사고 증가, 일상생활 수행 능력 저하, 우울 증가, 삶의 만족도 저하 등 부정적인 영향을 미친다[8].

시설 노인의 수면장애를 완화하기 위해 손마사지[9], 족욕요법[10], 발반사 마사지[11], 아로마요법[12], 이압요법[13], 웃음요법[14], 빛요법[A1-A3] 등 다양한 중재가 시행되었다. 국내외 노인에게 적용된 빛요법(light therapy)은 비교적 안전하고, 부작용이 적으며, 환자들도 쉽게 수용하므로 약물을 거부하는 환자이거나 약물이 허용되지 않는 환자에게 중요한 대체요법으로 이용된다[15]. 빛요법은 자연의 햇빛이나 인공 광선을 이용하여 인체의 생리적 변화를 유도하여 치료하는 방법으로 뇌의 신경 화학물질과 호르몬 분비를 조절하여 생체의 일주기 리듬을 조절한다. 즉, 낮 동안에 빛에 노출되면 세로토닌이 활성화되고, 밤에 멜라토닌 합성이 증가하여[16], 인지기능 및 수면장애, 우울, 초조행동 등을 완화할 수 있다[17]. 따라서 빛요법은 시설 노인의 수면장애 치료에 효과적이며 쉽고 간단히 적용될 수 있는 중재이다.

시설 노인을 대상으로 한 빛요법 연구들은 빛 노출 강도와 시간, 적용 시기, 중재 기간에서 차이를 나타내었다. 빛요법을 오전에 적용[18,19]하거나 오후에 적용[20]하기도 하고, 빛 노출 강도와 중재 기간도 다양하게 적용하였다[18-21]. 빛요법의 효과 또한 연구마다 차이가 있었다. 아침 시간대에 10,000 lux의 백색광을 30분간 노출하였을 때 수면의 질이 향상되었고[18], 6,000~8,000 lux의 빛을 아침 2시간 동안 2주간 적용한 연구에서는 야간 각성의 시간과 횟수가 감소하였다[19]. 반면, 오후에 2,500 lux의 빛을 2시간씩 4주간 적용한 연구에서는 유의한 변화가 나타나지 않았다[20]. 빛요법은 일주기 리듬의 개선에도 긍정적인 영향을 미쳤다[21]. 이처럼 개별 연구 결과 간의 차이가 존재하므로[16,22], 단일 연구 결과만으로는 시설 노인에 대한 빛요법의 전반적 효과를 일반화하기 어렵다. 따라서 시설 노인에게 적용된 빛요법 연구를 고찰하여 그 결과를 체계적으로 종합하여 통합적 근거를 제시할 필요가 있다.

지금까지 수행된 수면 개선을 위한 빛요법의 효과를 확인한 메타분석의 대상자는 성인[23], 불면증 환자[24], 인지기능 저하 대상자[25], 치매 환자[26], 암 환자[27] 등이었다. 대상자의 연령은 18세 이상[23], 60세 이상[25,26], 또는 전 연령층[24,27]으로 다양하였다. 또한 기존 메타분석 연구에 포함된 빛요법의 적용 환경은 외래를 포함한 지역사회, 혹은 지역사회와 시설, 병원을 모두 포함한 경우가 많았으며[23,26,27], 일부 연구에서는 수면장애에 대한 여러 보완·대체요법 중 하나로 빛요법을 함께 분석하였다[24].

따라서 본 연구에서는 65세 이상 시설 거주 노인을 대상으로 시행된 빛요법 연구를 체계적으로 고찰하고 메타분석을 수행하여, 빛요법이 수면에 미치는 효과를 확인하고자 한다. 이를 통해 시설 노인의 수면 개선 및 효과적인 빛요법 적용을 위한 객관적 근거를 제시하고자 한다.

### 2. 연구의 목적

본 연구의 목적은 시설 노인을 대상으로 한 빛요법이 수면에 미치는 효과를 보고한 실험연구를 체계적으로 고찰하여, 빛요법 중재의 특성을 확인하고 중재 특성에 따른 수면 효율성을 파악하는 것이다.

## 연구 방법

### 1. 연구 설계

본 연구는 빛요법 중재가 시설 노인의 수면 효율성에 미치는 효과를 규명하기 위한 체계적 문헌 고찰 및 메타분석 연구이다.

### 2. 문헌 선정 기준 및 배제 기준

문헌 선정은 PRISMA (Preferred Reporting Items for Systematic Review and Meta-analyses)에서 제시한 체계적 문헌고찰 지침에 따라 수행하였고[28], 구체적인 문헌 선정 조건은 PICO-SD (participants, interventions, comparisons, outcomes, study design)를 포함하였다. 연구 대상(participants)은 ‘국내·외 요양시설에 거주하는 65세 이상 노인’, 중재(interventions)는 ‘빛의 유형, 1회 적용 시 빛 노출시간, 빛의 강도, 빛 중재 기간, 적용 시기를 명시한 인공 빛요법’으로 하였으며, 비교 대상(comparisons)은 인공 빛요법을 제공하지 않은 무처치 대조군으로 하였다. 중재 결과(outcomes)는 ‘시설 노인 대상으로 빛요법의 수면 효율성을 측정한 결과를 포함하였고, 연구유형(study designs)은 무작위실험연구(randomized controlled trial, RCT)와 비무작위실험연구(non-randomized controlled trial, NRCT)를 포함하였다.

본 연구의 문헌 배제 기준은 단일 실험설계의 연구 및 단일 사례 연구, 조사연구, 질적연구 등과 같은 실험설계가 아닌 연구는 제외하였으며, 여러 중재를 포함한 연구, 의학적 수면장애 및 정신질환으로 치료를 받는 대상자가 포함된 연구, 시설 이외에 가정 간병, 외래 환자 진료소나 정신과 입원환자 시설을 포함하는 지역사회 대상 연구는 제외하였다. 한국어나 영어 이외의 언어로 출판된 문헌이거나 전문을 제공하지 않은 문헌도 제외하였다.

### 3. 문헌 검색 및 선정

본 연구의 문헌 검색은 출간 연도에 대해 제한을 두지 않았고, 전자 데이터베이스에서 제공하고 있는 연도부터 2022년 9월 4일까지 게재된 문헌들을 검색하였다. 검색 데이터베이스는 미국립의학도서관(National Library of Medicine)에 제시된 COSI (core, standard, ideal) 모델을 사용하였다[29]. 국내 데이터베이스는 한국교육학술정보원(Research Information Sharing Service, RISS), 국가과학기술정보센터(National Digital Science Library, NDSL), 한국의학논문데이터베이스(Korean Medical Database, KMBASE), 한국학술정보(Korean Studies Information Service System, KISS), 한국과학기술학회(Korea Institute of Science and Technology Information, KISTI), 국회도서관(National Assembly Library, NAL)의 데이터베이스에서 검색하였고, 국외에서 출판된 문헌은 MEDLINE (PubMed), EMBASE, Cochrane library, Cumulative Index to Nursing and Allied Health Literature (CINAHL), Proquest global 데이터베이스를 이용하였다. 검색어는 이전 연구들을 참고하여 PubMed의 MeSH 검색과 Embase의 Emtree내에서 주요 검색어, 유사어, 동의어, 자유어를 조사하여 검색어 간에 블리언 연산자(AND, OR)를 이용하고, ‘ALL Field’로 좀 더 포괄적인 검색을 하였다. 국내 데이터베이스에서 대상자는 ‘노인’ OR ‘시설 노인’ OR ‘장기요양시설’ 로 하고, 중재는 ‘빛요법’ OR ‘빛치료’ OR ‘빛중재’ OR ‘광요법’ 으로 하였으며, 결과로는 ‘수면’ OR ‘수면장애’ OR ‘수면의 질’로 검색하였고, 국내 문헌의 경우 전자적 검색만으로는 충분하지 않아, 추가로 수기 검색을 하였다.

국외 데이터베이스에서는 (“aged” OR “elderly” OR “older adult” OR “aging”) AND “facility” OR “Long-term care” OR “Long-term care facility” OR “Nursing home” OR “Nursing home resident”) AND (“light” OR “light therapy” OR “phototherapy” OR “bright light” OR “Expose Light” OR “light intervention”) AND (“Sleep” OR “Sleep Disorder” OR “Sleep Wake Disorders” OR “Insomnia”)로 하여 검색하였다.

**Figure 1.**
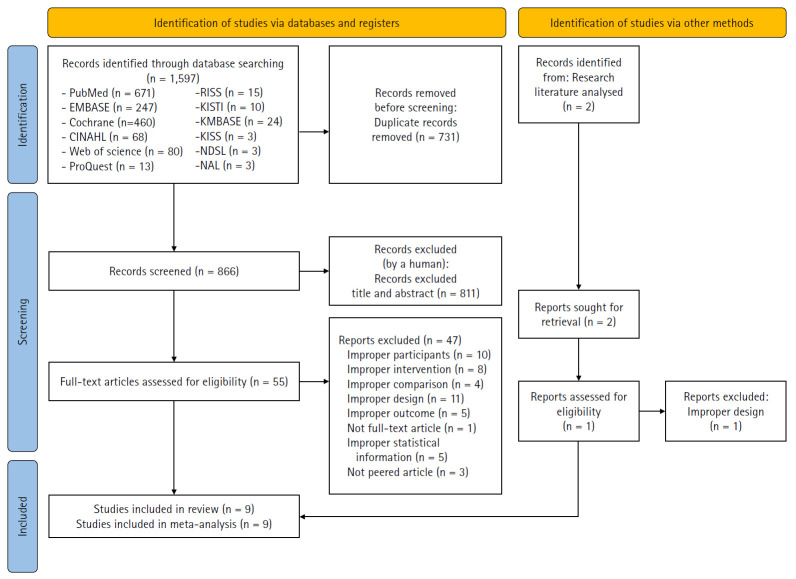
Preferred Reporting Items for Systematic Review and Meta-analyses (PRISMA) flow diagram of study selection process.

문헌 검색은 국립의학도서관에서 제시한 선정 기준인 COSI 모델에 근거하여 수행하였으며, 그 결과 총 1,597편의 문헌이 확인되었다. 이 중 중복된 731편을 제외한 866편의 문헌 중에서 제목과 초록을 검토하여 선정 기준에 부합하지 않는 811편을 제외하고 55편을 1차로 선별하였다. 이후 55편의 원문을 검토한 결과, 선정 기준과 배제 기준에 따라 47편을 제외하고 8편의 문헌이 최종 선정되었다. 수기 검색 결과 2편의 문헌이 추가로 확인되었다. 이 중 선정 기준과 배제 기준에 따라 1편을 최종 포함하여, 총 9편의 문헌을 체계적 문헌 고찰 대상에 포함하였다(Figure 1, Appendix 1). 검색된 문헌은 Microsoft Excel 2016 (Microsoft, Redmond, WA, United State)과 EndNote X20 (Clarivate, London, United Kingdom) 프로그램을 이용하여 분류하였다.

### 4. 자료 추출

본 연구에서는 최종 선정된 연구를 대상으로 연구정보(저자, 출판년도, 출판유형, 연구설계, 연구 수행 국가)를 파악하고, PICO-SD 기준에 따라 데이터를 코딩하였다. 연구 대상자의 특성(연령, 참여자 수)과 중재 특성(빛의 강도, 노출시간, 적용기간, 적용 시기, 빛의 유형, 사용 장치), 연구 결과변수(수면 효율성, 야간 수면시간, 야간 각성 횟수, 수면의 질) 값을 체계적으로 코딩하였다. 수면 효율성은 개인이 잠자리에 누워 있는 전체 시간 중 실제로 잠든 시간의 비율(%)이며[30], 선정된 연구에서는 활동기록기(actigraphy)를 사용하여 측정하였다. 주요 결과변수는 수면 효율성이었으며, 이차 결과변수로는 야간 수면시간, 야간 각성 횟수 및 수면의 질이었다. 주요 결과변수의 연구결과에 제시된 평균, 표준편차, 표본수를 효과분석에 이용하였다. 데이터 추출은 Microsoft Excel 2016 (Microsoft)을 이용하여 수행하였으며, 메타분석 경험이 있는 연구자 2인이 독립적으로 결과를 검토하고 상호 확인하는 과정을 거쳤다.

### 5. 문헌의 질 평가

선정된 문헌의 질 평가는 한국보건의료연구원(National Evidence-based Healthcare Collaborating Agency)의 체계적 문헌 고찰 지침에서 제시한 Cochrane 평가도구를 참고하여 수행하였다. RCT는 Risk of Bias 2.0 tool (RoB 2) [31]을, NRCT는 Risk of Bias Assessment tool for Non-randomized Studies of Interventions (RoBANS) [32]을 이용하여 평가하였다. 질 평가는 오류 평가의 위험을 최소화하고 한 명의 선입관에 영향을 받지 않도록 메타분석 경험이 있는 연구자 2인이 독립적으로 수행하였으며, 평가 과정에서 발생한 불일치는 충분한 논의를 통해 합의에 모두 도달하였다.

RoB 2는 ① 무작위배정 과정에서의 비뚤림(bias arising from the randomization process), ② 의도된 중재에서 벗어난 비뚤림(bias due to deviations from intended interventions), ③ 결과자료의 결측으로 인한 비뚤림(bias due to missing outcome data), ④ 결과 측정의 비뚤림(bias in measurement of the outcome), ⑤ 선택적 결과 보고에 의한 비뚤림(bias in selection of the reported result)의 5개 영역을 평가하였다. 각 문항은 ‘그렇다’, ‘아마도 그렇다’, ‘아마도 아니다’, ‘아니다’, ‘정보 없음’으로 평가하였으며, RoB 2의 알고리즘에 따라 비뚤림 위험을 ‘낮음(low risk of bias)’, ‘일부 우려(some concerns)’, ‘높음(high risk)’으로 판정하였다.

RoBANS는 6개 영역(domain)으로 구성되어 있으며, ① 대상자 선정(selection of participants), ② 교란변수(confounding variables), ③ 중재 노출 측정(measurement of exposure), ④ 결과 평가에 대한 눈가림(blinding of outcome assessments), ⑤ 불완전한 결과자료(incomplete outcome data), ⑥ 선택적 결과 보고(selective outcome reporting)에 의한 비뚤림을 평가하였다. RoBANS의 결과는 ‘낮음(low risk of bias)’, ‘명확하지 않음(unclear)’, ‘높음(high risk)’의 세 단계로 판정하였다.

### 6. 자료 분석

체계적 고찰에 포함된 문헌의 일반적 특성은 실수와 백분율, 평균과 표준편차 같은 기술통계로 분석하였다. 양적 합성이 가능한 경우에는 Review Manager version 5.4.1 (RevMan, The Nordic Cochrane Centre, Copenhagen, Denmark) 을 사용하여 메타분석을 실시하였다. 또한 출판 비뚤림(publication bias)과 민감도 분석(sensitivity analysis)은 R software version 4.1.2 (R Foundation for Statistical Computing, Vienna, Austria)를 이용하여 수행하였다.

#### 1) 효과 크기 산출

본 연구에서는 대상자의 특성 및 중재 환경이 다양하여 이질성이 존재할 것으로 예상됨에 따라, 평균 효과 크기(mean effect size)는 임의효과 모형(random effects model)을 사용하여 산출하였다. 수면 효율성은 효과 크기 계산에 필요한 평균, 표준편차, 표본수를 모두 제공하고 있었다. 여러 중재 집단을 사용한 연구는 빛요법만 적용한 것으로 산출하였고, 공유 집단인 대조군을 더 작은 표본크기의 두 개 이상의 그룹으로 나누어 독립적인 비교를 하였다[29]. 효과 크기 산출을 위해 개별 연구 자료에서 사전·사후 평균, 사전·사후 표준편차, 표본수를 이용하였다. 본 연구에서 각 개별 연구 결과의 효과 크기는 표준화된 평균 차이(standardized mean difference)를 산출하였고, 효과 크기의 통계치는 Cohen's d를 교정한 Hedges' ĝ를 사용하여 전체 효과 크기가 과대 추정되지 않도록 하였다. 마지막으로 산출된 효과 크기의 해석은 Cohen [33]의 기준에 따라 평균 효과 크기가 0.1~0.3 이하이면 작은 효과 크기, 0.4~0.7은 중간 정도의 효과 크기, 0.8 이상이면 큰 효과 크기로 해석하였고, 효과 크기의 95% 신뢰구간(confidence intervals, CI)을 통하여 각 연구의 효과 크기 신뢰구간이 제로(0) 값을 포함하는지에 따라 통계적 유의성을 검증하였다.

#### 2) 이질성 평가

본 연구의 효과 크기에 대한 이질성(heterogeneity) 판단을 위해 숲 그림(forest plots)을 통한 시각적 검사, 통계적 검정을 시행하였다. 통계적 검정방법으로는 전체 분산인 Q값을 산출하고 카이 제곱 검정을 시행하고, 총분산에 대한 실제 분산의 비율인 *I*^2^ (I-squared)값을 산출하였다. 일반적인 기준의 *I*^2^ 값은 25% 미만은 이질성이 적고, 30%~60%는 중간 정도의 이질성, 75% 이상이면 이질성이 매우 큰 것으로 해석하며, *I*^2^ 값이 50% 이상이고 카이 제곱 검정의 유의확률이 0.10보다 작으면 효과 크기의 이질성이 상당한 수준으로 평가한다[29].

#### 3) 출판 비뚤림 분석

본 연구에서는 수면 효율성에 대한 숲 그림과 깔때기 그림(Funnel plot)을 통해 출판 비뚤림(publication bias)의 비대칭 여부를 확인하였다. 분석에 포함된 연구 편수가 최소 10편 이상일 경우 Egger’s regression을 통한 통계적 검정이 권장되나[33,34], 본 연구에서는 연구 편수가 기준에 미치지 않아 Egger’s regression은 시행하지 않았다.

#### 4) 민감도 분석

빛요법의 수면 효율성에 미치는 효과의 일관성을 검증하기 위해 민감도 분석을 하였다. 각 연구를 하나씩 제외한 경우의 효과 크기를 모든 연구를 포함한 평균 효과 크기와 비교하여, 효과 크기의 안정성과 일관성을 확인하였다.

## 연구 결과

### 1. 문헌의 일반적 특성

본 연구의 분석에 포함된 문헌 9편의 출판연도는 2005년부터 2019년까지이며, 대상자의 특성을 살펴보며 치매를 진단받은 대상자인 연구가 6편, 치매는 아니지만 시설에 거주하는 노인을 대상자로 한 연구가 3편이었다. 연구 대상자의 평균 연령은 75~80세 3편(33.3%), 81~86세 6편(66.7%)이었다. 연구 참여자는 총 418명(빛요법 중재군 230명, 대조군 188명)이었다(Table 1).

### 2. 빛요법 중재의 특성

빛요법 중재의 특성을 살펴보면 인공 빛요법을 위한 장치로써 라이트 박스를 이용한 경우가 5편[A1,A2,A5,A6,A9]으로 가장 많았으며, 모자 형태의 라이트인 캡바이저 1편[A7], 천정형 조명 장치를 이용한 경우 2편[A3,A8], 라이트 튜브를 이용한 경우 1편[A4]이었다. 빛의 파장유형으로 백색광을 사용한 경우가 4편[A1-A3,A8], 청색광이 1편[A7], 청색광과 백색광을 사용한 경우가 2편[A4,A6], 보고되지 않은 경우 2편[A5,A9]으로 백색광을 가장 많이 사용하였다. 회당 빛 노출시간은 30분 4편[A5,A6,A7,A9], 60분 3편[A1,A2,], 8~12시간 3편[A3,A4,A8]으로 30분이 가장 많았고, 빛의 강도는 1,000~2,500 lux가 4편[A1,A2,A4,A6,A8]으로 가장 많았고 10,000~12,000 lux 연구가 2편[A5,A7]이었으며, 5,000 lux연구 1편[A9]과 567 lux 연구가 1편[A3]이었다. 빛요법을 적용한 시간은 오전[A1,A7,A9], 오후[A2,A5,A6], 낮 동안 종일 적용[A3,A4,A8]이 모두 각각 3편으로 동일하였다. 중재 기간은 1주부터 10주까지로 다양하였다(Table 1).

### 3. 문헌의 질 평가

**Figure 2.**
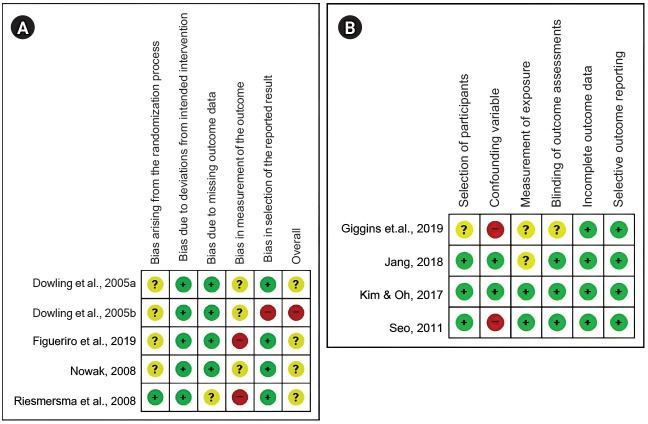
Quality appraisal. (A) randomized controlled trial, (B) non-randomized controlled trial.

RCT 연구 5편의 결과를 살펴보면 무작위배정 순서 과정에서 발생하는 비뚤림 위험은 4편이 ‘일부 우려’로 나타났으며, 1편은 ‘낮은 위험’으로 평가되었다. 의도된 중재에서 벗어난 비뚤림 위험은 5편의 연구는 모두 ‘낮은 위험’으로 평가하였다. 중재 결과자료의 결측으로 인한 비뚤림 위험은 4편이 ‘낮은 위험’, 1편이 ‘일부 우려’로 평가되었다. 중재 결과 측정의 비뚤림 위험은 3편이 ‘일부 우려’, 2편 ‘높은 위험’으로 판단하였다. 마지막 평가 항목으로 선택된 연구 결과 보고에 의한 비뚤림은 4편의 연구에서는 ‘낮은 위험’, 1편의 연구에서 ‘높은 위험’으로 평가하였다. 최종 전체 5개 영역에 대한 전반적인 비뚤림 위험 평가 결과로 최소 1가지 이상 ‘높은 위험’이 있는 연구는 3편으로 평가되었으나 전반적으로 ‘높은 위험’이 많은 연구는 없어 모든 연구를 분석 대상에 포함하였다(Figure 2-A).

NRCT의 연구 4편의 질 평가를 살펴보면, 부적절한 대상군 선정으로 발생한 비뚤림 위험에 대해 3편이 ‘낮은 위험’이었고, 1편의 연구에서 ‘명확하지 않음’으로 평가하였다. 교란변수 확인과 고려가 부적절하여 발생한 비뚤림 위험에서는 2편의 연구에서 ‘낮은 위험’, 2편의 연구에서는 교란변수에 대한 통제에 대해 언급하지 않고 있어 ‘높은 위험’으로 평가하였다. 부적절한 중재(노출) 측정으로 인해 발생한 실행 비뚤림은 2편의 연구에서 ‘낮은 위험’, 2편은 ‘명확하지 않음’으로 평가하였다. 결과평가에 대한 눈가림에서는 3편의 연구에서 ‘낮은 위험’이었고, 1편의 연구에서 ‘명확하지 않음’으로 평가하였다. 불완전한 자료에 대해 부적절하게 다루어 발생한 탈락 비뚤림 및 선택적 결과 보고로 인해 발생한 보고 비뚤림 항목은 4편 모두‘낮은 위험’으로 평가하였다(Figure 2-B). 무작위 실험연구 5편과 비무작위 실험연구 4편에 대한 질 평가 결과를 바탕으로 선정된 9편의 연구를 모두 메타분석에 포함하였다.

### 4. 빛요법이 수면에 미치는 효과 분석

#### 1) 수면 효율성의 효과 크기

**Figure 3.**
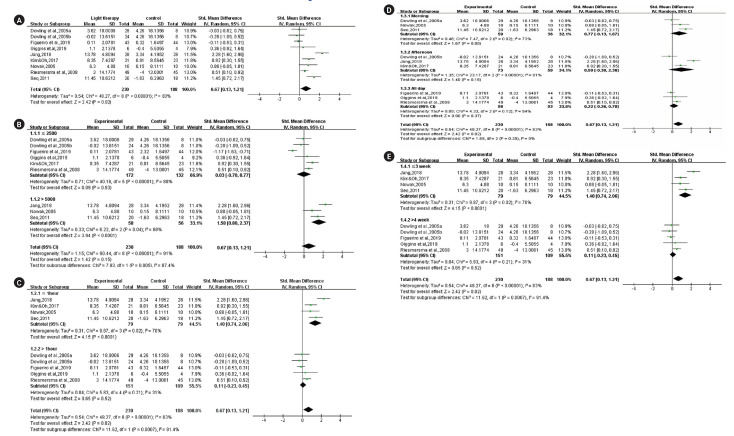
Forest plot of the effects of sleep efficiency by (A) light therapy, (B) light intensity, (C) light exposed duration, (D) timing of light exposure, (E) intervention period. SD = Standard deviation; IV = Inverse variance; CI = Confidence interval; Std = Standardized.

빛요법이 시설 노인의 수면 효율성에 미치는 효과 크기는 0.67 [95% CI: 0.13~1.21]로 Cohen의 기준에 따르면 중간 수준으로 나타났으며, 신뢰구간이 제로(0)를 포함하지 않아 통계적으로 유의하였다(Z = 2.42, *p* = .020). 또한 수면 효율성에 대한 이질성은 *I*^2^=83% (τ^2^ = 0.54, Q = 48.27, *p* < .001)로 높은 수준이었다(Figure 3-A).

#### 2) 중재 특성에 따른 수면 효율성의 효과 크기

본 연구에서는 빛요법이 수면 효율성에 미치는 효과를 비교하기 위해 하위집단 분석을 수행하였다. 빛요법의 효과 크기와 이질성은 빛의 강도, 노출시간, 적용 시기, 중재 기간 등의 특성에 따라 분석하였다.

##### (1) 빛의 강도

빛의 강도는 2,500 lux 이하와 5,000 lux 이상으로 범주화하였고, 5,000 lux 이상인 연구 3편은 이질성이 컸고(Q = 6.22, *p* = .040, *I*^2^ = 68%), 효과 크기는 1.58 [95% CI: 0.80~2.37]로 통계적으로 유의하여 수면 효율성 향상에 효과가 있었다(Z = 3.94, *p* < .001). 빛의 강도 2500 lux 이하인 연구 6편은 이질성이 컸고(Q = 40.15, *p* < .001, *I*^2^ = 88%), 효과 크기는 0.03 [95% CI: −0.70~0.77]로 통계적으로 유의하지 않았다(Z = 0.08, *p* = .930) (Figure 3-B).

##### (2) 빛 노출시간

일 회당 빛 노출시간의 경우 1시간 미만과 1시간 이상으로 범주화하였고, 1시간 미만인 연구 4편은 이질성이 컸으며(Q = 9.87 *p* = .020, *I*^2^ = 70%), 효과 크기는 1.40 [95% CI: 0.74~2.06]으로 큰 효과 크기를 나타내어 통계적으로 유의하였다(Z = 4.15, *p* < .001). 빛 노출시간 1시간 이상인 연구 5편은 이질성이 중간 이하(Q = 5.83, *p* = .210, *I*^2^ = 31%)로 확인되었고, 효과 크기는 0.11 [95% CI: −0.23~0.45]로 통계적으로 유의하지 않았다(Z = 0.65, *p* = .520) (Figure 3-C).

##### (3) 빛 적용 시기

빛의 적용 시기는 오전과 오후, 하루 종일로 범주화하였고, 오전 적용 3편은 이질성이 큰 정도(Q = 7.47, *p* = .020, *I*^2^ = 73%)로 확인되었고, 효과 크기는 0.77 [95% CI: −1.13~1.67]로 통계적으로 유의하지 않았으며(Z = 1.67, *p* = .090). 오후 적용 3편에서도 이질성이 컸고(Q = 23.17, *p* < .001, *I*^2^ = 91%), 효과 크기는 0.99 [95% CI: −0.39∼2.36]로 통계적으로 유의하지 않았다(Z = 1.40, *p* = .160). 빛을 하루 종일(오전 9시~오후 8시) 적용한 연구 3편에서 중간 정도의 이질성이(Q = 4.33, *p* = .120, *I*^2^ = 54%) 확인되었고, 효과 크기는 0.22 [95% CI: −0.26∼0.70]로 통계적으로 유의하지 않았다(Z = 0.90, *p* = .370) (Figure 3-D).

##### (4) 빛요법 중재 기간

빛요법 중재 기간은 3주 미만과 4주 이상으로 범주화하였고, 3주 미만인 연구 4편은 이질성이 큰 수준(Q = 9.87 *p* =.020, *I*^2^ = 70%) 이었고, 1.40 [95% CI: 0.74~2.06]의 큰 효과 크기를 나타내어 통계적으로 유의하였다(Z = 4.15, *p* < .001). 빛요법 중재기간이 4주 이상인 연구 5편은 이질성이 중간 이하(Q = 5.83, *p* = .210, *I*^2^ = 31%)이었고, 효과 크기는 0.11 [95% CI: −0.23~0.45]로 통계적으로 유의하지 않았다(Z = 0.65, *p* = .520) (Figure 3-E).

### 5. 출판 비뚤림

수면 효율성에 대한 메타분석에서 오류 존재 여부를 확인하기 위해, 표본오차가 작은 순으로 정렬한 숲 그림을 시각적으로 검토한 결과, 표본크기와 표본오차 간 일치가 관찰되어 소규모 연구 효과(small study effect)는 나타나지 않았다. 또한 깔때기 그림을 통해 모형의 대칭성을 검토한 결과, 전체적인 효과 크기 분포는 대체로 대칭을 이루었으나, 일부 연구가 깔때기 경계선 밖에 위치하였다(Appendix 2).

### 6. 민감도 분석

숲 그림 분석에서 개별 연구를 하나씩 제외했을 때 전체효과 크기의 변화가 0.39 [A5]~0.79 [A3] 범위로 제한적이며, 특정 연구 때문에 전체효과가 크게 변동하지 않았다. Baujat plot 분석 결과, Figueriro 등 [A3]의 연구가 전체효과 크기에 가장 큰 영향을 미쳤고, Jang [A5]의 연구는 전체효과 크기에 영향을 미치면서 전체 이질성에 상대적으로 크게 기여하고 있으나, 이 연구를 제외하여도 전체효과 크기의 방향성과 통계적 유의성에 영향을 주지는 않았다. 이러한 결과는 분석 결과가 비교적 안정적이며 일관됨을 지지한다(Appendix 3).

## 논의

본 연구에서는 시설 노인을 대상으로 한 빛요법 중재가 수면에 미치는 효과를 체계적으로 고찰하고, 최종 선정된 9편의 실험연구를 메타분석 하여 수면 효율성에 미치는 영향을 확인하였다. 분석 결과, 빛요법은 시설 노인의 수면 효율성을 향상시키는 긍정적 효과를 보였으며, 이는 빛이 생체리듬 조절에 중요한 역할을 한다는 기존의 보고[35]를 지지하였다.

시설 거주 노인은 평균 연령이 80세 이상으로 높고 치매 진단 비율 또한 높아, 인지기능 저하와 신체적 허약을 동반하는 경우가 많으며 이에 따라 자연광 노출이 제한될 수 있다. 이러한 환경적 요인은 수면 부족과 수면-각성 리듬 장애를 초래할 수 있는데, 본 연구에서 확인된 바와 같이 빛요법은 이러한 문제를 개선하는 데 유효한 중재로 여겨진다. 이는 빛이 망막-시상하부 경로를 통해 시교차상핵(suprachiasmatic nucleus)에 작용하여 일주기 리듬을 조절하고, 낮 동안 멜라토닌 분비를 억제하며 각성 체계를 활성화한다[35,36]는 생리학적 기전과도 부합한다.

본 연구에서 빛요법에 적용된 빛의 파장은 백색광을 사용한 경우가 4편으로 가장 많았다. 이는 대상자들이 전체 가시광선을 포함하는 광원(full spectrum light)인 백색광을 더 선호하며, 시중에서 구입할 수 있는 라이트 박스 대부분이 백색광을 사용하고 있기[37] 때문으로 여겨진다. 본 연구에서 사용된 빛의 강도는 500 lux에서 12,000 lux로 다양하였다. 빛의 강도는 균일한 측정 단위가 없으나 연구에서 일반적으로 사용되는 lux는 사람이 눈으로 인지하는 빛의 강도이다[38]. 본 연구에서는 5,000 lux 이상의 고강도 빛을 적용하면 노출시간이 30분으로 비교적 짧았고[A5-A7,A9], 1,200 lux 미만의 저강도 빛을 적용하면 노출시간이 10~12시간으로 상대적으로 길었다[A3,A4,A8]. 또한 중재 기간 역시 1주에서 10주까지 다양하였으며, 빛의 강도가 강할수록 노출시간은 짧고 중재 기간도 2주 이내로 적용되는 경향을 보였다. 수면 결과는 객관적인 도구를 이용하여 정확한 수면량의 측정이 필요하다. 인지기능이 저하된 고령의 노인인구에서 정확도를 높이면서 휴대가 편한 객관적 수면 측정 도구를 사용하면 결과의 신뢰도를 높을 수 있다[26]. 본 연구에서도 8편의 연구가 액티그래프를 통해 객관적 지표인 수면량을 측정해 사용하고 있어서 바람직하다고 여겨진다.

본 연구에서 빛요법이 수면 효율성에 미치는 전체 효과 크기는 0.67로 Cohen [33]의 기준에 따르면 중간 정도의 효과 크기였으며 통계적으로 유의하였다. 이러한 결과는 빛요법이 노인의 생체리듬을 조절하고 수면-각성 주기의 안정화에 이바지할 수 있음을 시사한다. 시설 노인을 대상으로 한 빛요법 메타분석이 기존에 거의 없어 직접 비교는 어려우나, 본 연구에서 관찰된 효과 크기는 성인을 대상으로 한 Maanen 등[23]의 메타분석 효과 크기 0.35보다 큰 편이었다. 반면, 치매 노인을 대상으로 한 Tan 등[26]의 연구에서는 효과 크기 0.17, 불면증 환자를 대상으로 한 Baglioni 등[24]의 연구에서는 ╶0.01로 유의한 효과를 나타내지 않아 본 연구와 차이를 보였다. Tan 등[26]은 연구 대상자 수가 수면 효율성과 수면시간의 효과 크기에 공변량으로 작용했으며, 효과 크기가 중간 이하였다는 점을 근거로 향후 적절한 표본크기를 확보한 엄격한 임상 연구가 필요하다고 제안하였다. 이러한 점은 시설 노인을 대상으로 시행한 빛요법 연구설계 시 충분한 표본 확보와 연구설계의 엄격성이 중요함을 시사한다.

중재 특성에 따른 분석 결과, 저강도 빛에서는 유의한 효과가 없었지만, 5,000 lux 이상 고강도 빛에서는 효과 크기가 1.58로 수면 효율성 향상에 유의한 효과를 보였다. 이러한 결과는 Fetveit 등[19]이 장기 요양시설 노인을 대상으로 6,000~8,000 lux 강도에서 적용한 결과와 유사하다. 노화에 따라 동공 크기가 감소하고 수정체 렌즈의 빛 투과율이 줄어 망막에 도달하는 조도가 낮아지기 때문에[39], 노인 대상 빛요법에서는 세팅된 조도와 실제 망막에 전달되는 빛의 양 간 차이가 발생할 수 있다. 따라서 빛의 강도가 높은 경우 더 긍정적인 효과가 나타난 것으로 해석된다. 또한, 빛 노출시간이 1시간 미만일 경우 효과 크기가 1.4로 수면 효율성 향상에 효과적이었는데, 이는 고령 노인을 포함한 불면증 성인을 대상으로 45분 동안 빛 노출 시 수면 개선을 확인한 선행 연구[40]와도 유사한 결과이다. 고령의 노인이나 치매가 있는 노인의 경우 주의 집중 시간이 짧아 장시간 빛에 노출되더라도 실제로 빛이 충분히 전달되지 않아 효과에 영향을 미칠 수 있다. 이러한 특성을 고려하여, 빛요법 제공 시 손을 움직이는 간단한 활동을 병행하면 피험자가 빛에 더 집중할 수 있어[41], 실제 임상 적용 시 이를 활용할 수 있다. 또한, 강한 빛 자극으로 인한 각막 및 망막 손상을 예방하기 위해 광원 스크린에 자외선 차단 필터를 설치하고, 치료 중에는 직접 광원을 바라보지 않도록 주의해야 한다[42]. 따라서 시설 노인의 경우 장시간 저강도 빛보다는 단시간 고강도의 빛요법이 더 효율적인 전략이 될 수 있으며, 대상자의 선호와 안전을 고려하여 빛요법을 설계하는 것이 중요하다.

빛요법을 적용한 시간을 오전, 오후, 하루 종일로 범주화하여 분석한 결과, 집단별 효과 크기는 유의하지 않았다. 본 연구에서 시간대별 효과성이 뚜렷하게 규명되지 않은 이유는 연구 수가 제한적이고 연구 간 이질성이 높았기 때문일 가능성이 있으나, Tan 등[26]의 메타분석에서도 빛의 적용 시기는 수면 효율성에 유의한 영향을 미치지 않는다고 보고하였다. 따라서 대상자의 선호도를 반영하여 빛요법 적용 시간을 설정할 경우, 일상의 리듬을 유지하면서 보다 편안한 수면을 유도할 수 있을 것으로 판단된다.

본 연구에는 몇 가지 제한점이 있다. 첫째, 분석에 포함된 연구 편수와 표본 수가 제한적이었으며, 연구설계와 중재 방법의 이질성이 존재하였다. 둘째, RCT 연구뿐 아니라 NRCT 연구가 포함되어 효과 크기가 다소 과대 평가될 수 있으며, 영어와 한국어로 언어를 제한하여 언어편향에 대한 우려가 있다. 따라서 향후 연구에서는 시설 노인을 대상으로 대규모 무작위 대조군 연구가 필요하며, 빛요법의 중재 강도, 노출시간, 기간, 적용 시점 등에 따른 최적의 중재 조건을 재규명할 필요가 있다. 그럼에도, 종합적으로 고려할 때 빛요법은 시설 노인의 수면 효율성을 향상시키는 유용한 중재 전략이 될 수 있다.

## 결론

본 연구 결과는 빛 노출 기회가 제한된 시설 노인을 대상으로 시행한 빛요법이, 비교적 간단하고 안전하며 비침습적인 방법으로 수면 효율성을 향상시키는 효과적인 중재임을 뒷받침하였다. 따라서 시설 내 간호 현장에서 빛요법을 활용할 것을 권장한다. 그러나 시설 거주 노인에게 빛요법을 적용할 때 일정 강도와 노출시간, 중재 기간 등을 고려할 필요가 있다. 궁극적으로 빛요법은 시설 노인의 수면장애를 완화하여 더 나은 일상생활을 가능하게 하고, 건강을 유지하는 데 도움이 될 것이다.

## Figures and Tables

**Table 1.** Characteristics of the Included Studies (N = 9)

No	Author (year)	Country	Study Design	Setting	Participants /MMSE^†^	Sex	Age (years)	Sample size (n)		Device	Intervention					Outcome (measures)
						M:W	(M ± SD)	EG	CG		Light source types	Intensity (lux)	Duration/session (hours)	Application	Intervention period/week	
A1	Dowling et al. (2005a)	USA	RCT	LTC facility	Dementia/**moderate~severe**	10:36	84.0 ± 1.0	29	17	Light box	White light	2,500	1	Morning	5 days a week for 10 weeks	Night time sleep
																Sleep efficiency
																Number of night awakenings (actigraphy)
A2	Dowling et al. (2005b)	USA	RCT	LTC facility	Dementia/moderate~severe	Not reported	84.0 ± 1.0	24	17	Light box	White light	2,500	1	Afternoon	5 days a week for 10 weeks	Night time sleep
																Sleep efficiency
																Number of night awakenings (actigraphy)
A3	Figueriro et al. (2019)	USA	RCT	LTC facility	Dementia/mild~moderate	16:30	EG 83.1 ± 6.2	43	44	Light box, table, floor	White light	567	10~12	6am~8pm	Daily for 4 weeks	Night time sleep
							CG 85.3 ± 7.7									Sleep efficiency (actigraphy), sleep quality (PSQI)
A4	Giggins et al. (2019)	Ireland	NRCT	LTC facility	Older residents (NR)	3:7	80.8 ± 6.6	6	4	Light tube	Blue light (AM)	1,200	11	9am~8pm	Daily for 4 weeks	Night time sleep
											white light (PM)					Sleep efficiency (actigraphy)
A5	Jang (2018)	Korea	NRCT	LTC facility	Dementia/moderate~severe	EG 10:18	EG 75.3 ± 8.1	28	28	Light box	Not reported	10,000	0.5	Afternoon	5 days	Night time sleep
						CG 16:12	CG 75.5 ± 6.8									Sleep efficiency (sleep record)
A6	Kim & Oh (2017)	Korea	NRCT	LTC facility	Institutionalized older adults (normal)	EG 12:9	70 ≧ 65: 17	21	23	Light box	Blue light	2,500	0.5	Afternoon	10 days	Sleep efficiency (actigraphy) sleep quality
						CG 6:17	71~80: 26									
						EG 12:10	≧ 81: 23	22			White light					
						CG 6:17										
A7	Nowak (2008)	USA	RCT	Nursing Home	Dementia mild~moderate	20 (W)	85.9 ± 6.2	10	10	Cap visor	Blue light	12,000	0.5	Morning	Daily for 2 weeks	Sleep efficiency
																Number of night awakenings (actigraphy)
A8	Riesmersma et al. (2008)	Netherlands	RCT	Nursing home	Older residents	EG 4:45	85.8 ± 5.5	49	45	Light	White light	1,000	8	10am~6pm	Daily for 6 weeks	Night time sleep
						CG 5:40				tube						Sleep efficiency (actigraphy)
A9	Seo (2011)	Korea	NRCT	LTC facility	Dementia mild~mode-rate	EG 4:16	EG 77.0 ± 5.9	20	18	Light box	Not Reported	5,000	0.5	Morning	3 weeks	Night time sleep
						CG 3:15	CG 79.2 ± 8.0									Sleep efficiency

MMSE = Mini Mental Status Examination; M = Men; W = Women; M = Mean; SD = Standard deviation; EG = Exercise group; CG = Control group; RCT = Randomized controlled trial; NRCT = Non-randomized controlled trial; LTC = Long term care; NR = Not reported; PSQI = Pittsburgh Sleep Quality Index. ^†^MMSE score: severe (≤ 10)-mild (< 25).

## Data Availability

The data that support the findings of this study are available from the corresponding author upon reasonable request.

## Appendix 1. List of Studies Included.

A1. Dowling GA, Hubbard EM, Mastick J, Luxenberg JS, Burr RL, Van Someren EJW. Effect of morning bright light treatment for rest-activity disruption in institutionalized patients with severe Alzheimer's disease. International Psychogeriatrics. 2005;17(2):221-236. https://doi.org/10.1017/S1041610205001584

A2. Dowling GA, Mastick J, Hubbard EM, Luxenberg JS, Burr RL. Effect of timed bright light treatment for rest-activity disruption in institutionalized patients with Alzheimer's disease. International Journal of Geriatric Psychiatry. 2005;20(8):738-743. https://doi.org/10.1002/gps.1352

A3. Figueriro MG, Plitnick B, Roohan C, Sahin L, Kalsher M, Rea MS. Effects of a tailored lighting intervention on sleep quality, rest-activity, mood, and behavior in older adults with Alzheimer disease and related dementias: a randomized clinical trial. Journal of Clinical Sleep Medicine. 2019;15(12):1757-1767. https://doi.org/10.5664/jcsm.8078

A4. Giggins OM, Doyle J, Hogan K, George M. The impact of a cycled lighting intervention on nursing home residents: a pilot study. Gerontology and Geriatric Medicine. 2019;5:1-6. https://doi.org/10.1177/2333721419897453

A5. Jang HY. Effects of light therapy on sleep and depression in elderly with dementia in a long-term care hospital [dissertation]. Daejeon: Daejeon University; 2018.

A6. Kim HJ, Oh JJ. The effect of bright light therapy (BLT) on sleep in institutionalized elderly (Application of blue and white light). 2017 Spring conference of KGS on social exclusion and quality of life of older persons; 2017 May 26; Seoul , Korea. The Korean Gerontological Society; 2017 May. p. 195-199.

A7. Nowak L. The effect of timed blue-green light on sleep-wake patterns in women with Alzheimer's disease [dissertation]. Detroit: Wayne State University; 2008.

A8. Riesmersma-van der Lek RF, Swaab DF, Twisk F, Hol EM, Hoogendijk WJG, Van Someren EJ. Effect of bright lightnd melatonin on cognitive and noncognitive function in elderly residents of group care facilities: a randomized controlled trial. The Journal of the American Medical Association. 2008;299(22):2642-2655. https://doi.org/10.1001/jama.299.22.2642

A9. Seo YS. Effects of light therapy on sleep disorders in long-term residential facilities elderly with dementia [dissertation]. Gyeongsan: Daegu Catholic University; 2011.

## Appendix 2. Publication bias.

Funnel plot for the effects on sleep efficiency.

## Appendix 3. Sensitivity analysis.

Forest plot and Baujat plot of sensitivity analysis. SMD = Standardised mean difference; CI = Confidence interval.
